# Outcomes of Prolonged Biliary Plastic Stent Dwell Time in Patients with Choledocholithiasis Undergoing ERCP Followed by Cholecystectomy

**DOI:** 10.3390/jcm14196869

**Published:** 2025-09-28

**Authors:** Tal Weiss, Oren Gal, Miri Elgabsi, Neev Tchernin, Veacheslav Zilbermints, Boris Kessel

**Affiliations:** 1 Department of General Surgery, Hillel Yaffe Medical Center, The Rapaport School of Medicine, Technion, Haifa 3525433, Israel; 2 Faculty of Medicine and Public Health, Tel Aviv University, Tel Aviv-Yafo 6997801, Israel; 3 Department of Gastroenterology, Hillel Yaffe Medical Center, The Rapaport School of Medicine, Technion, Haifa 3525433, Israel; 4 Rappaport Medical School, Technion, Haifa 3525433, Israel

**Keywords:** endoscopic retrograde cholangiopancreatography (ERCP), biliary stent, choledocholithiasis, cholecystectomy, recurrent biliary episodes

## Abstract

**Introduction:** ERCP with temporary biliary stenting followed by elective cholecystectomy and postoperative ERCP is commonly used to treat choledocholithiasis. While early stent removal (within 3–6 months) is generally recommended, some studies suggest that longer dwell time may not increase morbidity. This study aims to evaluate outcomes associated with prolonged stent dwell time of more than six months. **Methods:** We conducted a retrospective study of all patients who underwent ERCP with plastic biliary stent insertion, followed by elective cholecystectomy and postoperative ERCP at a single tertiary center between 2018–2024. Patients were divided into early-ERCP (≤6 months) and late-ERCP (>6 months) groups. The primary outcome was the rate of recurrent biliary episodes. Secondary outcomes included urgent postoperative ERCP, stent reinsertion, and the need for additional ERCP’s. **Results:** A total of 203 patients were included (mean age 58.3 ± 19.8 years). Thirty-one patients (15%) had a stent dwell time of more than six months. Demographic and presenting characteristics were comparable between groups, except for acute cholecystitis, which was more frequent in the early-ERCP group (18% vs. 3.2%, *p* = 0.034). Recurrent biliary episodes were significantly more frequent in the late-ERCP group (19.4% vs. 5.8%, *p* = 0.021), as were urgent postoperative ERCP (16.1% vs. 5.2%, *p* = 0.044), stent reinsertion (35.5% vs. 14.5%, *p* = 0.008), and additional ERCPs (38.7% vs. 15.7%, *p* = 0.006). **Conclusions:** Prolonged biliary stent dwell time beyond six months is associated with significantly higher rates of recurrent biliary episodes, urgent postoperative ERCP, postoperative stent reinsertion, and additional ERCP’s.

## 1. Introduction

Choledocholithiasis (bile duct stones) occurs in approximately 10–20% of patients with gallstones [1]. When untreated, it may result in obstructive jaundice, acute cholangitis, or gallstone pancreatitis, each carrying significant morbidity and mortality [2].

The recommended management of choledocholithiasis is guided by risk stratification and institutional expertise. The American Society for Gastrointestinal Endoscopy (ASGE) recommends that patients at high risk for choledocholithiasis—defined by the presence of a common bile duct (CBD) stone on imaging, ascending cholangitis, or both total bilirubin > 4 mg/dL and CBD dilation—should undergo endoscopic retrograde cholangiopancreatography (ERCP) as the preferred first-line diagnostic and therapeutic intervention for bile duct clearance. This should be followed by cholecystectomy to prevent recurrent biliary events, unless contraindicated by comorbidities or patient preference [2].

For patients with intermediate risk, further evaluation with endoscopic ultrasound (EUS), magnetic resonance cholangiopancreatography (MRCP), or intraoperative cholangiography is recommended before proceeding to ERCP or surgery [2].

Surgical alternatives, such as laparoscopic common bile duct exploration (LC-BDE), can be performed as a single-stage procedure during cholecystectomy and have demonstrated comparable rates of stone clearance and adverse events to two-step strategies involving ERCP and cholecystectomy [3]. Randomized trials indicate that, although overall outcomes are similar, the two-step approach is often associated with longer hospital stays, particularly when ERCP is performed preoperatively [3]. In younger patients, single-stage LCBDE is frequently preferred because it avoids papillotomy and enables definitive treatment in one session [4]. Other two-step strategies, such as laparoscopic cholecystectomy with intraoperative cholangiography followed by postoperative ERCP for positive findings, show similar efficacy; however, choledochotomy carries a notable risk of bile leak. More recently, selective strategies guided by intraoperative cholangiography have been associated with shorter hospitalizations by avoiding unnecessary procedures in patients with a low prevalence of choledocholithiasis [2]. 

When ERCP is employed for the treatment of choledocholithiasis, an endoscopic sphincterotomy is routinely performed as an inherent part of the procedure. The ASGE specifically recommends sphincterotomy as the standard technique for bile duct stone removal, since it provides reliable access to the common bile duct and significantly improves the likelihood of successful and complete stone extraction. Furthermore, sphincterotomy has been shown to decrease the risk of recurrent biliary events when it is followed by a definitive cholecystectomy [2]. However, it should not be regarded as a substitute for surgical removal of the gallbladder and is generally reserved as a sole therapy only for patients who are considered poor surgical candidates or medically unfit for operative intervention [5]. 

A plastic biliary stent is often placed during the index ERCP, particularly in patients with acute cholangitis or significant obstructive jaundice, as it provides immediate biliary drainage and decompression, and may also facilitate delayed stone extraction when initial clearance is unsuccessful [6,7]. A plastic biliary stent is also considered beneficial when the interval to subsequent cholecystectomy cannot be definitively assured at the time of the index ERCP, due to either patient- or system-related limitations. Less frequently, stent placement may be indicated in the context of anesthesiological constraints requiring urgent termination of the procedure, or in patients whose clinical condition necessitates minimizing procedure duration.

Current guidelines issued by both the European Society of Gastrointestinal Endoscopy (ESGE) and the American Society for Gastrointestinal Endoscopy (ASGE) recommend that plastic biliary stents should be exchanged at regular intervals, ideally every three months, in order to minimize the risks of stent occlusion, cholangitis, and other procedure-related complications [2,6]. In line with these recommendations, the majority of institutions worldwide schedule a follow-up ERCP for stent retrieval within approximately three to six months after the index procedure [8]. Primary ERCPs performed urgently for biliary drainage in cases of acute cholangitis, hemodynamic instability or severe inflammation during the acute phase may occasionally be limited in their ability to achieve complete ductal clearance at the initial intervention. In such cases, post-cholecystectomy ERCP also serves to confirm biliary clearance and manage any residual stones or sludge [9,10].

Several articles demonstrate that adherence to the recommended timelines for biliary stent exchange in real-world practice is often variable and influenced by multiple factors. This issue became particularly evident during the COVID-19 pandemic, which disrupted the scheduling of endoscopic procedures, including timely removal of plastic biliary stents [8,11,12]. Despite this, data on the outcomes of prolonged stent dwell time beyond six months remain limited. While a number of studies have reported an increased risk of complications—most notably acute cholangitis, stent occlusion, and recurrent biliary obstruction [13]—others have not demonstrated a clear association between extended stent dwell time and increased morbidity [12,14,15]. One study suggested that although maintaining a biliary stent in place for more than 12 months may increase the likelihood of developing cholangitis, this strategy can still be considered an acceptable management option in carefully selected high-risk patients who are not suitable for more definitive interventions [16]. However, this study specifically evaluated a cohort of patients who were not eligible for repeated sessions of endoscopic lithotripsy or for surgical procedures, thereby limiting the application of its findings to the broader population of patients with choledocholithiasis.

Other studies have referred to these long-dwelling biliary stents, which were not intentionally left in place for delayed retrieval, as “forgotten biliary stents.” These are often defined as stents retained for longer than 12 months [17,18,19]. Forgotten biliary stents have been associated with adverse outcomes, including stent occlusion, stent migration, and cholangitis. While these complications have also been reported to occur earlier after stent placement, they are generally observed at lower rates during the initial months. [12,20]. The extent and timing of these complications remain incompletely understood, and an optimal time frame for safe stent removal has yet to be clearly defined.

One potential mechanism for these complications is the development of a stent–stone complex, wherein the stent acts as a nidus for stone formation regardless of the presence of choledocholithiasis at the time of the initial ERCP [21,22]. Over time, these stones may encase or adhere to the stent, complicating retrieval and increasing the risk of infection or obstruction. This phenomenon has been particularly associated with stenting durations exceeding 301 days [21].

Given the clinical implications of prolonged stent dwell time, this study aimed to evaluate the outcomes associated with plastic biliary stent dwell times exceeding six months in patients undergoing ERCP followed by elective cholecystectomy for the management of bile duct stones.

## 2. Materials and Methods

This retrospective study was designed to evaluate the clinical outcomes associated with prolonged biliary stent dwell time beyond six months in patients undergoing ERCP followed by elective cholecystectomy for the treatment of bile duct stones.

### 2.1. Patients’ Selection

The study population included all patients who underwent ERCP with plastic biliary stent insertion followed by cholecystectomy and postoperative ERCP at Hillel Yaffe Medical Center between January 2018 and November 2024. Patients were excluded if they had pancreaticobiliary malignancies, benign biliary strictures, or a non-naïve major papilla, as well as those who did not ultimately undergo cholecystectomy.

### 2.2. Ethical Aspects

The study protocol was reviewed and approved on 24 November 2024 by the Institutional Review Board of Hillel Yaffe Medical Center (HYMC-0076-22).

### 2.3. Study Outcomes

The primary aim of the study was to evaluate and compare the risk of recurrent biliary episodes after stent insertion between patients whose stent dwell time was less than six months and those in whom the dwell time exceeded six months. Recurrent biliary episodes were defined as the occurrence of symptomatic biliary obstruction, acute cholangitis, or stent migration, each supported by corresponding laboratory abnormalities and imaging findings, and requiring either hospitalization or therapeutic intervention. These recurrent events were systematically assessed across three distinct time intervals: the preoperative period, the postoperative period, and the post-stent extraction period.

The secondary aims of the study were to compare, between the two groups, the incidence of urgent postoperative ERCP, the frequency of restenting during postoperative ERCP, and the overall requirement for additional ERCP procedures. In order to minimize bias related to variability in follow-up duration, the maximum follow-up period for all patients was standardized and set according to the longest stent dwell time observed within the cohort.

### 2.4. Study Design and Definition

Data for the analysis were retrieved from the hospital’s electronic medical records. Collected variables included demographic characteristics, such as age and gender as well as clinical parameters at presentation, including laboratory and imaging findings.

The diagnosis at first admission was defined as the primary indication for further biliary evaluation and included choledocholithiasis, acute cholangitis, biliary pancreatitis, or acute cholecystitis. Choledocholithiasis was considered the diagnosis at first admission when patients were assigned relevant ICD-9 codes (574.21, 574.51, or 574.91), regardless of subsequent diagnostic confirmation by additional imaging or endoscopic evaluation. Complicated disease was defined as a primary diagnosis of choledocholithiasis, biliary pancreatitis, or cholangitis at the time of initial presentation. The decision to pursue further biliary evaluation with endoscopic ultrasound (EUS), magnetic resonance cholangiopancreatography (MRCP), or endoscopic retrograde cholangiopancreatography (ERCP) was made primarily in accordance with contemporary guideline recommendations. Patients presenting with clear indications for ERCP—such as ascending cholangitis, the presence of visible bile duct stones on primary imaging, or persistent jaundice with a bilirubin level exceeding 4 mg/dL in the context of a dilated common bile duct (CBD > 6 mm)—were directed to undergo ERCP as the initial diagnostic and therapeutic step. In contrast, patients with borderline or less definitive features underwent further evaluation with EUS or MRCP, and ERCP was subsequently performed only when choledocholithiasis was confirmed. An additional indication for ERCP was the demonstration of CBD filling defects on cholangiography, which was performed through a previously placed cholecystostomy tube. In our institution, patients treated with a cholecystostomy for acute cholecystitis routinely undergo cholangiography prior to tube clamping or removal, as well as before definitive cholecystectomy.

At our institution, the target is to schedule the postoperative ERCP for stent removal within 3 months of the index ERCP and no later than 6 months whenever feasible. There were no protocolized criteria to intentionally maintain stents beyond 6 months; dwell times >6 months reflected real-world constraints (intercurrent illness or exacerbation of existing chronic health conditions, social/logistic barriers and adherence, endoscopy capacity limits, and COVID-19-related postponement of elective activity). Stent dwell time was defined as the interval from the index ERCP (stent placement) to the postoperative ERCP (stent removal). Patients were categorized as early-ERCP (dwell time ≤ 6 months) or late-ERCP (dwell time > 6 months).

### 2.5. ERCP Procedure

The biliary stent routinely used in our institution is the Boston Scientific Flexima biliary stent (Amsterdam type), which measures 7 cm in length and 10 Fr in diameter. During the index ERCP, the standard strategy in our institution is to perform an endoscopic sphincterotomy in all cases to facilitate bile duct access and effective stone clearance, in accordance with published ASGE recommendations. In cases where prophylaxis for post-ERCP pancreatitis was indicated, a pancreatic stent measuring 5 cm in length and 5 Fr in diameter, without a leading barb, was inserted. This particular type of pancreatic stent was selected for its self-migrating properties, which allow for spontaneous passage and minimize the need for subsequent retrieval.

### 2.6. Postoperative Management and Follow-Up

For elective laparoscopic cholecystectomy, patients received a single prophylactic antibiotic dose within 60 min before skin incision to prevent surgical-site infection; postoperative antibiotics were not routinely continued unless there was documented cholangitis or intraoperative contamination requiring targeted therapy. Multimodal analgesia was used (paracetamol ± non-steroidal anti-inflammatory drugs, with short-course opioid rescue if needed). Oral intake was initiated on the day of surgery and advanced as tolerated, with early ambulation encouraged. Discharge was typically planned for postoperative day 1 if the patient was afebrile, hemodynamically stable, tolerating diet, mobilizing independently, and had adequate pain control with oral medications, with no concern for bile leak or other complications. Outpatient follow-up was scheduled at ~4 weeks, and the postoperative ERCP for stent removal was arranged at discharge.

### 2.7. Statistical Analysis

Statistical analysis was carried out using R software, version 4.3.1 (The R Foundation for Statistical Computing, Vienna, Austria) in conjunction with RStudio 2024.12.1+563. Categorical variables were summarized and reported as frequencies with corresponding percentages, while continuous variables were expressed either as means with standard deviations or as medians with interquartile ranges (IQR, 25th–75th percentiles), depending on the distribution of the data. Normality of continuous variables was evaluated using the Shapiro–Wilk test and inspection of Q–Q plots. Nominal data were compared using the χ^2^ test or Fisher’s exact test, as appropriate. Continuous variables were analyzed with the Mann–Whitney U test. A *p*-value of ≤0.05 was considered to indicate statistical significance. Multivariate regression analysis was performed to identify predictors of recurrent biliary episodes, and the results were reported as odds ratios (ORs) with corresponding 95% confidence intervals (CIs). Kaplan–Meier analysis was used to estimate the cumulative incidence of recurrent biliary episodes over time. Time zero was the date of the index ERCP (stent placement). The event was the first recurrent biliary episode (symptomatic biliary obstruction, acute cholangitis, or stent migration) requiring hospitalization or therapeutic intervention. Patients were censored at the earliest of: (i) the date of postoperative ERCP + 7 days (to capture immediate post-extraction events), (ii) last clinical contact documented in the EHR, or (iii) the standardized maximum follow-up of 966 days. Cholecystectomy itself did not constitute a censoring event.

## 3. Results

A total of 203 patients underwent ERCP with plastic biliary stent insertion followed by subsequent cholecystectomy and postoperative ERCP at a single medical center between 2018 and 2024 (Table 1). The mean age was 58.3 ± 19.8 years. The distribution of sex within the cohort was relatively balanced, with females accounting for 52.7% of the study population. The majority of patients underwent laparoscopic surgery, which was successfully completed in 192 cases (94.6%). Open surgery was performed in 5 patients (2.5%), while laparoscopic procedures required conversion to open surgery in 6 cases (2.9%).

The most common indication for ERCP was choledocholithiasis, observed in 87 patients (42.9%), followed by acute cholangitis in 53 patients (26.1%), acute cholecystitis in 32 patients (15.8%), and biliary pancreatitis in 31 patients (15.3%).

The primary imaging modality at presentation was ultrasound (US), used in 58% of the patients, followed by computed tomography (CT) in 24.4%, and both modalities in 17.6%. Based on initial laboratory and imaging findings, upfront ERCP was performed on 65% of the patients. Ten patients (4.9%) underwent MRCP, 56 patients (27.6%) underwent EUS, and 5 patients (2.5%) were diagnosed with choledocholithiasis by means of cholangiography performed through a cholecystostomy tube.

At presentation, 71 patients (31.0%) had CBD stones on primary imaging, 131 patients (64.5%) demonstrated a dilated CBD, and 82 patients (40.4%) showed elevated bilirubin levels.

The median interval between the initial and postoperative ERCP was 113 days (IQR 93–161). The median time from the index ERCP to surgery was 38 days (IQR 14–57), while the median time from surgery to postoperative ERCP was 78 days (IQR 52.5–107). Eight patients had an ERCP-to-ERCP interval longer than 12 months, with the maximum interval reaching 966 days. This duration served as the reference for the total follow-up period for all patients.

In the Late-ERCP group (*n* = 31), 22 patients had delays attributed to compliance/social factors or COVID-19–related disruptions. Three patients had surgical issues contributing to the delay before the second ERCP (one cholecystoduodenal fistula that was diagnosed during cholecystectomy; two postoperative collections), and six experienced exacerbations of comorbid conditions that postponed ERCP.

Overall, 16 patients (7.9%) had a recurrent biliary episode during the follow-up period. Of these, 9 events occurred in the interval between surgery and the postoperative ERCP, 5 episodes were recorded within one week of stent retrieval, and 2 episodes developed later during follow-up. Fourteen patients (6.9%) required an urgent postoperative ERCP instead of a scheduled ambulatory procedure. Thirty-six patients (17.7%) underwent biliary stent insertion during the postoperative ERCP due to incomplete biliary clearance, and 39 patients (19.2%) required additional ERCP.

Of the total cohort, 172 patients had a stent dwell time of less than six months (Early-ERCP group), while 31 patients had a stent dwell time exceeding six months (Late-ERCP group) (Table 2). There were no significant demographic differences between the groups. However, acute cholecystitis as the initial diagnosis was significantly more common in the Early-ERCP group (18% vs. 3.2%, *p* = 0.034), whereas complicated presentations such as cholangitis, biliary pancreatitis, and choledocholithiasis were more frequent in the Late-ERCP group. Despite these clinical differences, imaging and laboratory findings—including dilated common bile duct, presence of CBD stones, and elevated bilirubin—were similar between the groups.

The rate of recurrent biliary episodes was significantly higher in the late-ERCP group compared to the early-ERCP group. (19.4% vs. 5.8%, *p* = 0.021). Similarly, the incidence of urgent postoperative ERCP, postoperative biliary stent insertion, and the requirement for additional ERCP procedures were all significantly more frequent in the late-ERCP group than in the early-ERCP group (16.1% vs. 5.2%, *p* = 0.044; 35.5% vs. 14.5%, *p* = 0.008; and 38.7% vs. 15.7%, *p* = 0.006, respectively). Notably, when the late-ERCP group was further stratified into two subgroups according to stent dwell time—those with dwell times of less than 12 months and those with dwell times exceeding 12 months—the corresponding rates of recurrent biliary episodes were 13.0% and 37.0%, respectively.

Multivariate regression analysis was performed to identify predictors of recurrent biliary episodes (Table 3). Demographic characteristics, the presence of complicated primary diagnoses, and undergoing open surgery were not significantly associated with the occurrence of these events. In contrast, a stent dwell time longer than six months was found to be independently associated with a higher risk of recurrent biliary episodes, with an odds ratio (OR) of 4.42 and a 95% confidence interval (CI) of 1.30–14.52

**Figure 1:**
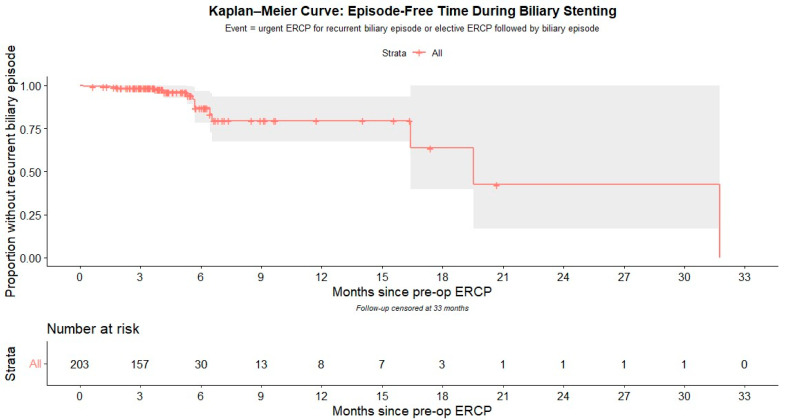
Kaplan—Meier Curve: Episode-Free Time During Biliary Stenting.

Kaplan–Meier survival analysis was used to depict the cumulative risk of recurrent biliary episode based on the duration of biliary stent retention, as shown in Figure 1.

The probability of remaining free of a recurrent biliary episode was 98.5% at 3 months (95% CI 96.8–100), 87.0% at 6 months (95% CI 78.2–96.7), and 79.6% at 12 months (95% CI 67.6–93.6), corresponding to cumulative incidences of 1.5%, 13.0%, and 20.4%, respectively. Median follow-up by reverse Kaplan–Meier was 3.75 months (≈114 days).

## 4. Discussion

ERCP followed by subsequent cholecystectomy is a widely adopted approach for the management of bile duct stones and their associated complications [3]. During the index ERCP, biliary stents are frequently inserted to ensure adequate drainage and to facilitate further biliary clearance at a later stage [2,6]. In such cases, a postoperative ERCP is generally required for stent removal. This second, post-cholecystectomy ERCP also provides an additional opportunity to achieve complete bile duct clearance in situations where this was not fully accomplished during the initial intervention [9,10]. 

The reported incidence of recurrent CBD stones after cholecystectomy with initial duct clearance varies considerably across the literature, ranging from 2% to 21%, depending on the study series [23,24,25,26,27,28]. The most severe manifestation of this complication is acute suppurative cholangitis, which may progress to sepsis and, if untreated, result in death. Early post-cholecystectomy choledocholithiasis (typically within two years) is generally attributed to retained stones—either undetected preoperatively or spilled from the gallbladder during surgery [29]. In contrast, late recurrences are more often considered to be de novo stones formed within the bile ducts [30]. 

The wide variability in CBD stones recurrence rates is thought to reflect the influence of multiple patient-related, anatomical, and procedural factors. Patient factors associated with higher recurrence include advanced age (particularly >65–70 years). Anatomical risk factors include a markedly dilated CBD (≥15 mm), the presence of multiple or large stones (≥2 stones or ≥10 mm in size), cholesterol stone composition, sharp bile duct angulation (<120–145°), and the presence of a periampullary diverticulum. Procedural contributors to recurrence include repeated ERCP sessions and the use of endoscopic mechanical lithotripsy [25,26,28,31,32,33,34].

Importantly, the presence of biliary stents has been implicated as a meaningful contributing factor in the development and recurrence of bile duct stone formation. Emerging evidence suggests that biliary stents may promote stone formation via a process known as the “stent–stone complex”. Prolonged stent retention is thought to contribute to biliary stasis, bacterial colonization, and precipitation of bile components, resulting in stones that encase or adhere to the stent [21]. Recent studies have reported that the recurrence rate of common bile duct stones after ERCP may be higher than after surgical common bile duct exploration—ranging from 6–21% versus 2–14%, respectively [23,24]. These findings further support the hypothesis that biliary stents may increase the risk of de novo stone formation.

In our cohort, 16 patients (7.9%) experienced a recurrent biliary episode, defined as the occurrence of symptomatic biliary obstruction, acute cholangitis, or stent migration that necessitated hospitalization or therapeutic intervention. Notably, while other studies often classify these events separately—such as distinguishing cholangitis, stent occlusion, and stent migration—we elected to analyze them collectively as a single entity, as they all essentially represent stent-related morbidity. Furthermore, in clinical practice, it can be challenging to clearly differentiate between these entities. For example, patients with clinically significant stent obstruction or migration are frequently managed as presumed cholangitis, even when they do not strictly meet the diagnostic criteria outlined in the Tokyo guidelines. A larger cohort would likely be required to allow for sufficient statistical power to identify specific risk factors for each individual complication.

No patient experienced a recurrent biliary episode during the interval between ERCP and cholecystectomy, a finding that can likely be attributed to the relatively short waiting period between procedures, with a median of 38 days (IQR 14–57). A total of nine recurrent biliary events were documented in the interval between surgery and the postoperative ERCP. An additional five episodes occurred within one week of stent retrieval, and it is not unlikely that these were related to the recent endoscopic intervention, possibly reflecting a technically difficult procedure. Finally, two biliary episodes occurred later during follow-up, which likely coincides with the well-known phenomena of biliary episodes occurring in post cholecystectomy patients after ERCP. This phenomenon remains a significant clinical concern, mostly during the first few years with recurrence rate as high as 4–24% [35].

A longer stenting duration was significantly associated with these events, supporting the hypothesis that de novo stone formation may partly explain post-cholecystectomy cholangitis or obstructive jaundice in patients with biliary stents. Notably, the multivariate regression analysis did not demonstrate any statistically significant association between the occurrence of recurrent biliary episodes and initial presentation with complicated disease, defined as choledocholithiasis, cholangitis, or biliary pancreatitis. This finding further supports the assumption that the primary cause of biliary events in these cases is stent related recurrent stone formation, rather than residual or retained stones resulting from inadequate biliary clearance at the time of the initial procedure.

In a prospective study that included 78 patients who were treated with plastic biliary stents for common bile duct stones, performing regular stent exchanges at fixed intervals of every three months was shown to significantly reduce the incidence of cholangitis when compared with an on-demand exchange strategy [13]. In accordance with these findings, the European Society of Gastrointestinal Endoscopy (ESGE) recommended in 2019 that plastic stents be exchanged within three to six months in order to minimize the risk of stent-related complications [6]. However, more recent studies—mostly conducted during the COVID-19 pandemic—have reported that stent retrieval after longer dwell durations can be performed safely, without a marked increase in adverse outcomes [12,14]. In our cohort, approximately one in five patients (19.4%) with stents retained beyond six months had a recurrent biliary episode, whereas only 5.8% of patients in the early-ERCP group developed such an event. Notably, previous studies included heterogeneous patient populations, comprising both benign and malignant etiologies, as well as patients eligible for cholecystectomy and those managed with repeated ERCP and stenting alone. In contrast, our study focused exclusively on patients undergoing ERCP followed by cholecystectomy as definitive treatment for choledocholithiasis.

Currently, an increasing number of ERCPs are performed in patients without a naïve major papilla, primarily for stent exchange or removal. A recent study from a tertiary care center reported that only 25% of ERCPs were index procedures, with the remaining 75% performed as follow-ups [27]. In our study, we demonstrated that a stenting duration exceeding six months was significantly associated with a higher rate of restenting during the second ERCP, as well as an increased likelihood of requiring additional ERCP. This may reflect persistent biliary stones or technical challenges that necessitate placement of a new protective stent, thereby leading to the need for further sessions to achieve definitive bile duct clearance.

This study adds to the literature suggesting that prolonged plastic stent dwell time (>6 months) is associated with increased complications. Given the higher event rates observed with dwell times >6 months, it is reasonable for centers to aim for stent removal within approximately 3 months and to generally avoid retention beyond 6 months when feasible. 

This study has several limitations. First, we lack definitive documentation of bile duct clearance prior to cholecystectomy. At our institution, the goal is to achieve complete biliary clearance during the index ERCP whenever feasible. However, even under optimal conditions, ERCP has inherent diagnostic limitations due to its reliance on two-dimensional imaging, which may fail to detect small or residual stones. As already mentioned, even when clearance appears to be complete, a plastic biliary stent is sometimes placed to maintain bile duct patency when the timing of the subsequent cholecystectomy cannot be definitively assured at the index ERCP due to patient- or system-related limitations, or based on the indication for ERCP. A second post-cholecystectomy ERCP is then performed to retrieve the stent and confirm bile duct clearance. Given that 26% of the patients in our cohort presented with cholangitis, it is plausible that some underwent cholecystectomy without complete bile duct clearance. Nonetheless, the comparable baseline characteristics and cholangitis rates between the Early- and Late-ERCP groups suggest that incomplete initial clearance is unlikely to fully account for the observed differences in outcomes.

Second, the Early-ERCP (*n* = 172) and Late-ERCP (*n* = 31) groups were uneven, reflecting our institutional practice to remove stents within ≤6 months whenever feasible. The small Late-ERCP group may limit statistical power and increase the risk that observed associations reflect chance or unmeasured confounding. We therefore emphasize effect sizes with 95% confidence intervals rather than significance alone. Larger prospective studies with standardized event adjudication are needed to confirm our findings and refine risk estimates.

Third, ERCP-related adverse events (e.g., post-ERCP pancreatitis, bleeding, perforation, cholangitis) and intra-procedural difficulties at stent removal (e.g., fragmentation, occlusion, unplanned repeat ERCP) were not systematically captured in the source records and therefore could not be quantified by group. As a proxy, we counted biliary events within 7 days after stent removal as stent-related, which may under- or over-estimate true procedure-related complications. Future prospective studies should include standardized complication reporting.

Finally, we lacked comprehensive data on patients’ comorbidities and socioeconomic status. At our institution, the target is to schedule the postoperative ERCP for stent removal within 3 months of the index ERCP and, when feasible, no later than 6 months. There were no protocolized criteria to intentionally maintain a stent beyond 6 months; prolonged dwell time reflected real-world constraints—including intercurrent illness, exacerbation of existing chronic health conditions, social or logistical barriers and adherence, limited endoscopy capacity, and COVID-19–related delays of elective services. It is plausible that patients with prolonged biliary stenting represented a sicker population or had more complex social and medical circumstances than those who underwent timely follow-up; this, in turn, could confound outcomes by introducing selection bias. Nevertheless, the approximately 20% rate of recurrent biliary episodes observed in the Late-ERCP group represents a clinically significant adverse outcome, with potential for major morbidity and even mortality—particularly in medically vulnerable patients. Further research into the predictors of delayed follow-up ERCP could help identify at-risk patients and guide targeted interventions. In selected cases—such as patients with a prolonged interval between initial ERCP and cholecystectomy, or those with barriers to follow-up—it may even be reasonable to consider performing the postoperative ERCP during the same hospital admission to reduce the risk of complications associated with delayed stent removal.

## 5. Conclusions

Prolonged biliary stent dwell time beyond six months is associated with significantly higher rates of recurrent biliary episodes, urgent postoperative ERCP, postoperative stent reinsertion, and the need for additional ERCP procedures. Our findings support targeting plastic stent removal within 3 months and avoiding dwell times >6 months whenever feasible.

## Figures and Tables

**Table 1:** Demographic/clinical characteristics and outcomes of patients who underwent ERCP with temporary biliary stenting followed by elective cholecystectomy and postoperative ERCP (*N* = 203).

Median Age in Years (± SD)	58.3 ± 19.8
Female gender	107 (52.7%)
Diagnosis at first admission	
Acute cholecystitis	32 (15.8%)
Cholangitis	53 (26.1%)
Biliary pancreatitis	31 (15.3%)
Choledocholithiasis	87 (42.9%)
Primary imaging modality	
US	112 (58%)
CT	47 (24.4%)
Both	34 (17.6%)
Further evaluation	
MRCP	10 (4.9%)
EUS	56 (27.6%)
Cholangiography through a cholecystostomy tube	5 (2.5%)
Surgical approach	
Laparoscopic surgery	192 (94.6%)
Open surgery	5 (0.2%)
Laparoscopic surgery converted to open surgery	6 (0.6%)
CBD stones on primary imaging	71 (35%)
Wide CBD on primary imaging	131 (64.5%)
Elevated bilirubin on first admission	82 (40.4%)
Median Time between ERCPs in days (IQR)	113 [93–161]
Median Time from ERCP to surgery in days (IQR)	38 [14–57]
Median Time from surgery to post-op ERCP in days (IQR)	78 [52.5–107]
Recurrent biliary episode	16 (7.9%)
Urgent postoperative ERCP	14 (6.9%)
Stent reinsertion during post-op ERCP	36 (17.7%)
Third ERCP	39 (19.2%)

SD, standard deviation; ERCP, endoscopic retrograde cholangiopancreatography; US, ultrasound; CT, computed tomography; MRCP, magnetic resonance cholangiopancreatography; EUS, endoscopic ultrasound; CBD, common bile duct; IQR, interquartile range.

**Table 2:** Comparison between Early-ERCP group (stenting period less than 6 months) and Late-ERCP group (stenting period more than 6 months).

	Early-ERCP Group (*N* = 172)	Late-ERCP Group (*N* = 31)	*p* Value
Age (±SD)	58.1 ± 19.4	59.3 ± 22.5	*p* = 0.791
Female gender	93 (54.1%)	14 (45.2%)	*p* = 0.436
Diagnosis at first admission			
Acute cholecystitis	31 (18%)	1 (3.2%)	*p* = 0.034
Cholangitis	44 (25.6%)	9 (29%)	*p* = 0.663
Choledocholithiasis	72 (41.9%)	15 (48.4%)	*p* = 0.557
Biliary pancreatitis	25 (14.5%)	6 (19.4%)	*p* = 0.586
Wide CBD on primary imaging	107 (63.3%)	24 (77.4%)	*p* = 0.153
Bile duct stones on primary imaging	58 (34.3%)	13 (41.9%)	*p* = 0.421
Elevated bilirubin on first admission	71 (43%)	11 (35.5%)	*p* = 0.552
Recurrent biliary episode	10 (5.8%)	6 (19.4%)	*p* = 0.021
Urgent postoperative ERCP	9 (5.2%)	5 (16.1%)	*p* = 0.044
Stent reinsertion during post-op ERCP	25 (14.5%)	11 (35.5%)	*p* = 0.008
Additional ERCP	27 (15.7%)	12 (38.7%)	*p* = 0.006

SD, standard deviation; ERCP, endoscopic retrograde cholangiopancreatography; CBD, common bile duct.

**Table 3:** Multivariate Logistic Regression: Predictors of Recurrent Biliary Episodes.

Variable	OR (95% CI)	*p*-Value
Age (per year)	0.99 (0.96–1.02)	*p* = 0.4502
Female gender	0.67 (0.22–1.94)	*p* = 0.4567
Complicated diagnosis	0.53 (0.14–2.54)	*p* = 0.3707
Prolonged stenting	4.42 (1.3–14.52)	*p* = 0.0141
Open surgery	0.73 (0.03–5.11)	*p* = 0.7865

## Data Availability

The data presented in this study are available on request from the corresponding author.

## Abbreviations

The following abbreviations are used in this manuscript:

ASGE	American Society for Gastrointestinal Endoscopy
CBD	Common bile duct
CI	Confidence interval
CT	Computed tomography
EUS	Endoscopic ultrasound
ERCP	Endoscopic retrograde cholangiopancreatography
ESGE	European Society of Gastrointestinal Endoscopy
IQR	Interquartile range
LCBDE	Laparoscopic common bile duct exploration
MRCP	Magnetic resonance cholangiopancreatography
OR	Odds ratio
US	Ultrasound
